# Results of Wedgeless Distal Femoral Osteotomy for the Treatment of Genu Valgus Deformity

**DOI:** 10.7759/cureus.31500

**Published:** 2022-11-14

**Authors:** Gur Aziz Singh Sidhu, Harjot Kaur, Islam Mubark, Ahmed Alwadia, Mohamed Nagy, Neil Ashwood

**Affiliations:** 1 Trauma and Orthopaedics, University Hospitals of Derby and Burton NHS Foundation Trust, Burton, GBR; 2 Anaesthesia, Queen Elizabeth Hospital, London, GBR; 3 Trauma and Orthopaedics, Cairo University Hospitals - Kasr Alainy, Cairo, EGY

**Keywords:** wedgeless "v" osteotomy, tibiofemoral angle, intermalleolar distance, distal femoral osteotomy, bostman's score, intermalleolar distance (imd), tibiofemoral angle (tfa), valgus deformity

## Abstract

Introduction: Coronal plane knee deformities are common disorders affecting adolescents. Valgus deformities (tibiofemoral angle (TFA) > 12-15 degrees and intermalleolar distance (IMD) > 10 cm) often require corrective osteotomy and a wedgeless "V" distal femoral osteotomy is a good treatment option for such deformities.

Materials and methods: Thirty adolescent patients (13-17 years) with valgus deformities were included. Patients with severe collateral ligament instability, subluxation, and sagittal plane deformity > 15 degrees or genu valgum due to tibial deformity were excluded. Preoperative clinical (Bostman's knee score, IMD, and knee-flexion test) and radiological evaluations were done. The surgery (wedgeless distal femoral V osteotomy) was performed and stabilized with two Kirschner wires (K-wires). Postoperative clinical and radiological parameters were recorded including complications.

Results: The preoperative TFA was 20.23 ± 3.63 degrees, which reduced to 5.5 ± 0.73 at six months postoperatively. The preoperative IMD was 12.45 ± 2.2 cm, which reduced to 1.63 ± 0.32 cm at six months. The mean mechanical axis deviation (MAD) and lateral distal femoral angle (LDFA) were recorded as 2.8 ± 0.39 and 87.7 ± 0.83, respectively, and the differences were statistically significant from preoperative values. The Bostman score was 26.2 ± 1.79 at three months and 29.47 ± 0.9 at six months. The complications included infection in two patients, a hypertrophic scar in one patient, and common peroneal neuropraxia in one patient.

Conclusion: Wedgeless distal femoral osteotomy with K-wire fixation is a viable option for correction of genu valgus deformity with potential advantages of minimal blood loss, no leg length discrepancy, non-rigid fixation, and early union as compared to other treatment options.

## Introduction

Coronal plane deformities around the knee are common disorders affecting older children and adolescents. In developing countries like India, nutritional rickets is the leading cause of these deformities [1]. These may originate from either the distal femur or proximal tibia but most often genu valgus deformity originates in the distal femur [2]. In genu valgus, there is increased anatomic tibiofemoral angle (TFA > 12 degrees) and intermalleolar distance (IMD > 8 cm) with lateralization of the mechanical axis. A decrease in mechanical lateral distal-femoral angle (mLDFA) indicates that genu valgus deformity originates in the distal femur [3]. Often, patients with genu valgus deformity have medial hamstring weakness, which may cause lateral tibial rotation [4]. The causes of genu valgus in adolescents can be idiopathic, post-traumatic, metabolic, neuromuscular, or post-infectious [1-3]. If the TFA of more than 15 degrees or an IMD of 10 cm persists after the age of 10 years, surgical treatment is often warranted. In such cases, surgery is performed to restore normal mechanical-axis alignment of the joint [5,6]. Different types of corrective osteotomies of the distal femur have been described in the literature, including tibial medial closing wedge osteotomy, lateral femoral opening wedge osteotomy, medial femoral closing wedge osteotomy, dome osteotomy, and wedgeless “V” osteotomy [7-12]. Corrective osteotomies can be performed to correct both congenital malalignment and post-traumatic deformity.

Patients with TFA > 12-15 degrees and IMD > 10 cm are considered for corrective osteotomy. Lateral femoral open wedge osteotomy causes lengthening of the limb, stretching of the iliotibial band, delayed union with more restricted weight bearing, non-union, a requirement of bone grafting, and secondary translational deformity of the osteotomy fragments. It becomes unstable if a large wedge is removed [7]. Prolonged healing, injury to neurovascular structures (popliteal), and iliotibial band stretching due to the translation of osteotomy are potent disadvantages [8]. It can become unstable if a large wedge is removed, as is required in the correction of severe valgus. In such situations, fixation with a blade plate is required, which is technically difficult because of the open growth plate and may lead to early physeal closure and growth disturbances [9,10]. Dome osteotomy leads to complete axial realignment without translation of osteotomy fragments and limb length alteration. It is chosen instead of a wedge osteotomy to avoid limb shortening that occurs in closing wedge osteotomy. Complications of dome osteotomy are non-union, failure of fixation, and peroneal neuropathy [11].

The wedgeless V-shaped distal femoral osteotomy has potent advantages of a small surgical incision, less operating time and incidence of bleeding intraoperatively, and no leg length discrepancy. Moreover, the entire surgery is done without entering the knee joint, which reduces the incidence of stiffness, adhesions, and intra-articular infection. The apex of “V” that is embedded in the metaphyseal bone gives additional inherent stability to the osteotomy. Moreover, the alignment can be adjusted after surgery during cast application, and a cancellous bone graft can be obtained from the proximal “V” fragment, which can be used and results in the early union at the osteotomy site [12]. The purpose of this study was to evaluate the functional and radiological outcomes and to check for complications, if any, of wedgeless distal femoral osteotomy in adolescents (13-17 years) for genu valgus deformity at the knee.

## Materials and methods

The study was conducted from 2016 to 2018 after obtaining informed written consent from the patients and clearance from the institutional review board (IRB). Thirty adolescent patients aged 13-18 years with TFA > 15 degrees or IMD > 10 cm were included. Patients with severe instability at the knee, subluxation, sagittal plane deformity > 15 degrees, or genu valgus causes due to tibial deformity were excluded.

**Figure 1:**
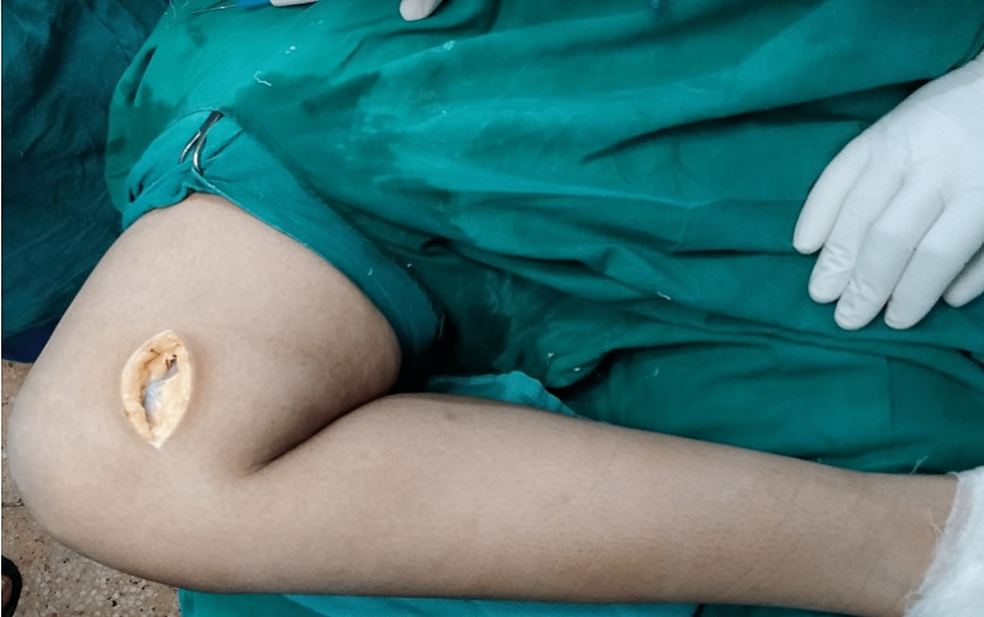
Medial incision in the figure of four position

Preoperative evaluation and clinical evaluation (Bostman's knee score, IMD, and knee-flexion test) were done for all patients. X-rays (anteroposterior and lateral views) of the knee with hip and ankle (single film) in the standing position were performed and TFA, mechanical axis deviation (MAD), and mLDFA were recorded. The operation was performed under general anesthesia (GA) with the patient supine on the operating table under tourniquet control. The knee was flexed to 60 degrees by keeping the limb in the figure of four position (lateral malleolus touching the opposite leg). A medial longitudinal skin incision (6-8 cm) was done from the adductor tubercle toward the medial joint line, as shown in Figure 1. The incision was carried deep through the superficial and deep fascia.

**Figure 2:**
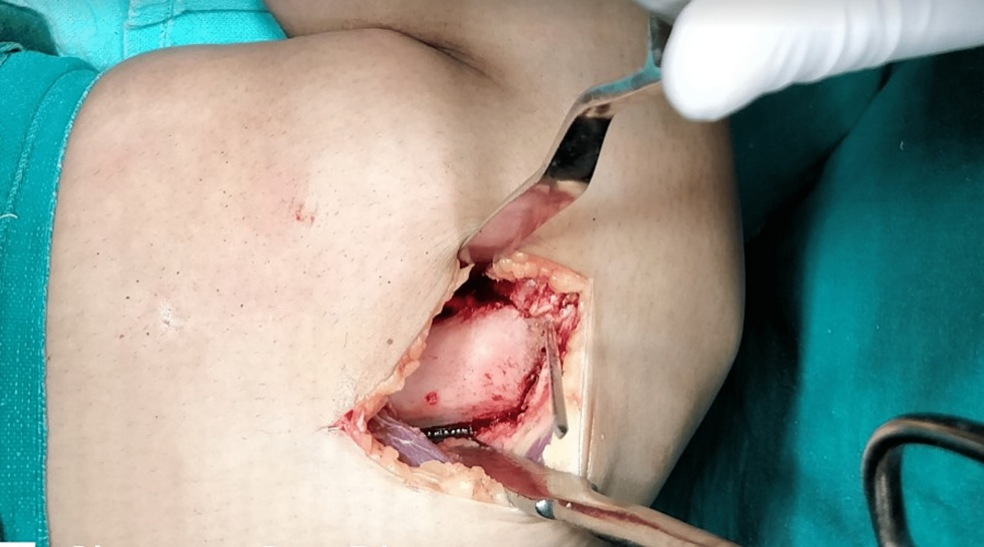
Distal femoral osteotomy with the apex of V at adductor tubercle

The vastus medialis muscle was elevated anteriorly along with the periosteum to expose the femoral metaphysis. The osteotomy was V-shaped in the frontal plane with the apex situated above the adductor tubercle (Figure 2).

**Figure 3:**
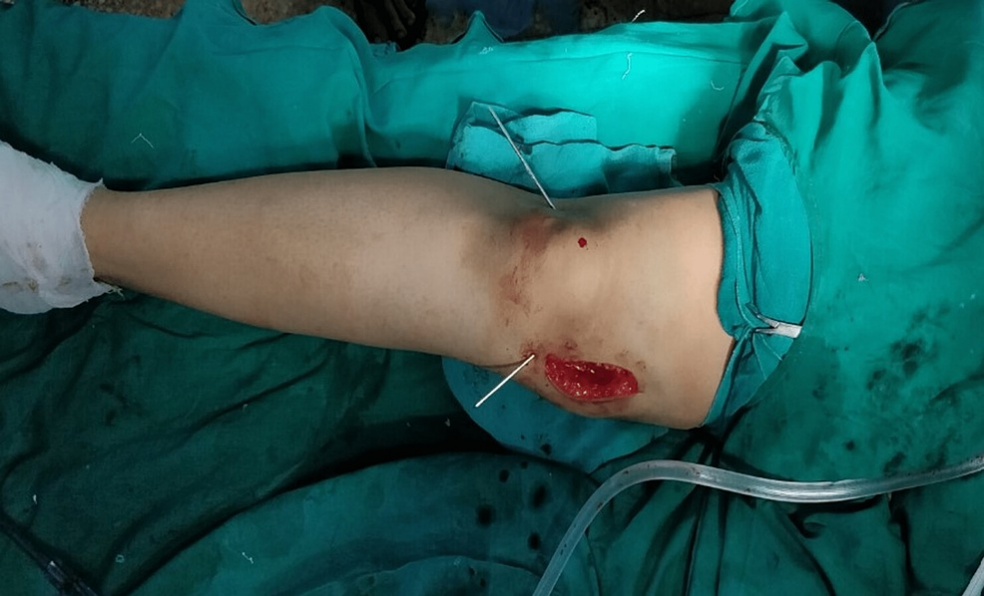
Corrected deformity fixed with K-wires

Before performing the osteotomy, a 2 mm Kirschner wire (K-wire) was introduced 2-3 mm proximal to the adductor tubercle, parallel to the distal femoral condyles. The position was checked under an image intensifier. The osteotomy cut was started with an oscillating saw and completed with an osteotome. Subsequently, the knee was extended, and the deformity was corrected with gentle manual varus force without traction. A further cut of 2 mm bone from the proximal anterior and posterior segment of the medial cortex was made to correct the deformity. The lateral cortex was opened in a hinged manner and was stabilized by soft tissues in our cohort. The alignment was repeatedly checked in extension and the degree of medial cortex penetration was closely monitored. Two crossed K-wires were passed from the medial and lateral sides to stabilize the osteotomy (Figure 3).

**Figure 4:**
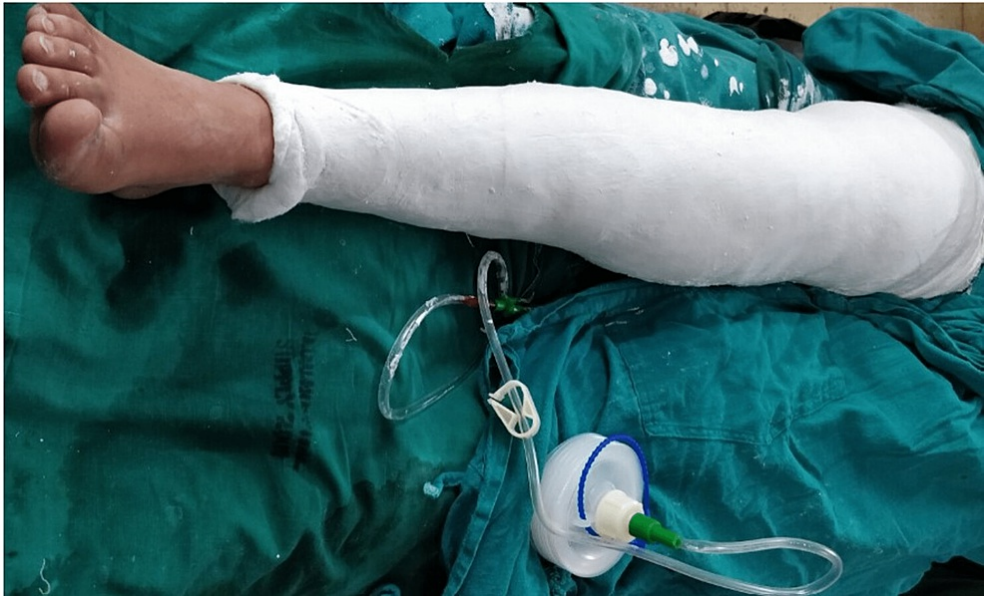
Knee immobilization cast

A suction drain was placed at the osteotomy site for 24 hours and the wound was closed in layers. Sterile dressing was done and a high groin cast extending from groin to ankle was applied (Figure 4).

Postoperatively, intravenous antibiotics were given for three days, and sutures were removed after two weeks. After the cast and K-wires removal at six weeks, the patient was kept on a non-weight-bearing crutch walk for three weeks and range of motion knee exercises were started, which progressed to full weight-bearing walking at 12 weeks. The patients were followed up weekly for one month, fortnightly for two months, and monthly for six months after surgery. The parameters of Bostman’s score were evaluated at six weeks, three months, and then at six months and radiological assessment of the knee was done at six, 12, and 24 weeks. Any complications during the follow-up period were recorded and appropriately managed.

Categorical variables were presented in numbers and percentages and continuous variables were presented as means ± SD and medians. The normality of the data was tested by the Kolmogorov-Smirnov test. Quantitative variables were compared using Wilcoxon signed rank test. The data were entered in a Microsoft Excel spreadsheet (Microsoft Corporation, Redmond, WA) and analysis was done using Statistical Package for Social Sciences (SPSS) version 21.0 (IBM Corp., Armonk, NY).

## Results

Our 30-patient cohort included 18 females (60%) and 12 males (40%). The mean age of the patients in our cohort was 15.4 ± 1.54 years (13-17 years). We had 14 cases of left-side deformity, 12 cases of right-sided deformity, and four cases with bilateral deformity. The majority of patients with genu valgus deformity were post-rachitic (18 cases) and idiopathic (10 cases), and two cases were with post-traumatic deformity.

The mean preoperative TFA was 20.23 ± 3.63 degrees, which reduced to 2.9 ± 0.92 degrees at six weeks and 5.5 ± 0.73 degrees at six months. This reduction in TFA was statistically significant (p < 0.0001). The mean preoperative IMD was 12.45 ± 2.2 cm, which reduced to 1.2 ± 0.34 cm at six weeks and 1.63 ± 0.32 cm at six months. This difference was statistically significant (p < 0.0001).

The mean MAD reduced to 2.8 ± 0.39 degrees at six months postoperatively from a preoperative value of 18.96 ± 2.62 degrees (p < 0.001). The mean lateral distal femoral angle (LDFA) postoperatively was 87.7 ± 0.83 degrees, which rose from a preoperative value of 76.9 ± 3.33 degrees, and this difference was statistically significant.

We encountered a few complications, which are depicted in Table 1. A limb length discrepancy of 1 cm was present in one patient, as it was present preoperatively as well.

**Table 1:** Complications in our cohort CPN: common peroneal nerve.

Complications	No. of patients
CPN neuropraxia	1
Hypertrophic scar	1
Infection	2
Total	4

**Figure 5:**
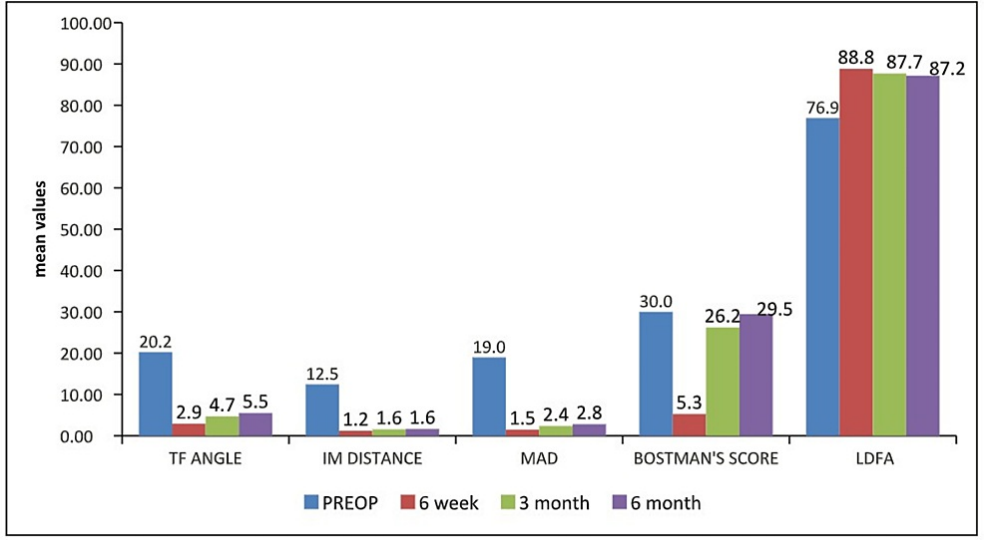
Summary of preoperative and postoperative measurements in our cohort TF: tibiofemoral; IM: intermalleolar; MAD: mechanical axis deviation; LDFA: lateral distal femoral angle.

The preoperative mean Bostman’s score was 30 ± 0, which was 5.27 ± 0.98 at six weeks, 26.2 ± 1.79 at three months, and 29.47 ± 0.9 at six months (Table 2). The summary of patient characteristics, duration of the cast, and radiological union as well as weight-bearing status are shown in Table 3. A bar diagram compared preoperative and postoperative measurements in our cohort (Figure 5).

**Table 2:** Bostman’s knee score in our cohort

Bostman's knee score	6 weeks	3 months	6 months
Excellent (28-30)	6.66%	36.66%	96.67%
Good (20-27)	33.33%	53.34%	3.33%
Satisfactory (<20)	60.01%	10%	0%

**Table 3:** Summary of characteristics recorded FWB: full weight bearing.

Parameter	Mean ± SD	Median	Min-Max
Age (years)	15.4 ± 1.54	15	13-18
Duration of cast (weeks)	6.27± 0.58	35	25-55
Radiological union (weeks)	9.47 ± 0.78	9	9-12
FWB status (weeks)	12.2 ± 0.61	12	12-14

## Discussion

Coronal plane deformities frequently cause knee pain, gait changes, instability, and the development of secondary changes such as early osteoarthritis due to changes in knee biomechanics. Hence, surgical correction is recommended nowadays to restore knee biomechanics [13]. Supracondylar closing wedge osteotomy is the most widely practiced osteotomy for the correction of genu valgus. The osteotomy is usually stabilized by a blade plate or a plate and screws. Despite having satisfactory outcomes, potential disadvantages include a large incision, rigid fixation, no adjustment postoperatively, and the need for a second surgery for implant removal [14]. Wedgeless "V" shape distal femoral osteotomy requires small surgical exposure and less operating time, has minimal bleeding when done under a tourniquet, has inherent stability at the osteotomy site (penetration of apex in distal metaphysis), and has reduced incidence of knee stiffness as this technique is done without entering the knee joint. Further advantages include no limb length discrepancy, early union at the osteotomy site, and non-rigid fixation, which allows postoperative adjustments [12,14].

Gupta and colleagues in their study assessed the outcome of using “V” osteotomy fixed with a customized “L” buttress plate [14]. The majority (95.7%) had excellent outcomes as judged by Bostman's score [14]. The authors reported an improvement in IMD from 13.8 to 1.5 cm, 23.5°-6.1° in clinical TFA, 22.2°-5.1° in radiological TFA, and 79.2°-89.1° in LDFA. We reported similar results of improvement as far as clinical and radiological outcomes were concerned. However, none of our patients required a second surgery for implant removal as we used K-wires in our study group. Moreover, the average blood loss in our study was 10 ml, as compared to 125 ml in a study conducted by Gupta et al. [14]. Another advantage of our technique was a gradual correction, which could be undertaken in the postoperative period due to some flexibility at the osteotomy site with K-wires rather than a rigid implant. We still encountered one case of common peroneal nerve (CPN) neuropraxia, which was resolved with conservative treatment postoperatively.

Literature reported studies in which the author used K-wires from the medial side for stabilization of osteotomy and achieved excellent results [15-17]. It was our observation that such a technique could be suitable for young children; however, it would be difficult to hold the osteotomy in place with single-sided wires in adolescents. We reported excellent results (96.67%) with crossed wires and felt it was a better mode of implant used for securing fixation. Some authors reported shorter periods (six weeks) of a radiological union at osteotomy sites as compared to our cohorts (average of nine weeks) [14]. We observed that this difference was mainly because of the difference in age in the two cohorts (age 10-12 years vs. 13-18 years in our study). An important complication regarding the slippage of distal femoral epiphysis was observed in a study conducted by Agarwal et al. [18]. The probable reason could be an excessive intraoperative force that could have crushed the distal metaphyseal cancellous bone leading to such a slip. However, in our cohort, all patients had fused distal physis, and hence, this complication was not encountered.

We observed one case of infection similar to the study conducted by Agarwal et al., which was controlled with oral antibiotics [18]. Also, we encountered one patient with common peroneal neuropraxia, who had correction of TFA from 33 degrees preoperative to 5 degrees postoperatively, which resolved with conservative treatment with a foot drop splint. Unfortunately, we had one case of CPN neuropraxia, but we still advise gradual correction of the valgus deformity with TFA of more than 25 degrees. The benefit of gradual correction is not possible with rigid implants and additional common peroneal nerve decompression is required if full correction is done in a single sitting. Thus, another incision on the lateral aspect of the knee is required leading to another scar that might not be acceptable to young female patients.

Our study had a few limitations despite excellent results. Firstly, it was a prospective study where a comparison between the techniques or implants could not be reported. However, our results were comparable to other studies done. Moreover, we could not predict the effects of osteotomy and changes in joint biomechanics during adult life.

## Conclusions

The wedgeless V-shaped distal femoral osteotomy with K-wire fixation could be a viable option for the correction of genu valgus deformity. The potential advantages included small incision, minimal blood loss, no leg length discrepancy, decreased chances of knee stiffness, non-rigid fixation, and early union.
